# Supplementary material for: Dynamic transcriptomic profiles of zebrafish gills in response to zinc supplementation
Source: BMC Genomics. 2010 Oct 11;11:553. doi: 10.1186/1471-2164-11-553 (PMC3091702; doi:10.1186/1471-2164-11-553)
Supplement: Additional file 2 — Interactive Direct Interaction Network representing the molecular interactions between zinc, copper, iron, calcium and proteins encoded by transcripts changed by zinc supplementation. Mini web-site containing index.html and hyperlinked pages in subdirectory describing a Direct Interaction Network automatically generated based on curated interactions contained within the proprietary PathwayArchitect database. Ovals represent proteins and the circles symbolize metal ions. Objects are coloured by their abundance in zebrafish at the time-point they were significantly different from the control is a scale from -4 fold (dark green) to +4 fold (dark red). Where significant differences were found at more than one time-point, the colour overlay shows expression at the first instance. Dark blue squares denote 'binding', and light blue squares 'expression'; green squares stand for 'regulation', green diamonds for 'metabolism', and green circles for 'promoter binding'. Arrow heads indicate directionality of the interaction where annotated. All nodes and edges can be further interrogated by selecting the relative area of the image. [file 1471-2164-11-553-S2.zip › PathwayArchitect Zn xs DIN/394825.html]

# BINDING:

|  |  |
| --- | --- |
| Type | BINDING |
| Effect | None |


---

|  |  |
| --- | --- |
| Score | 0 |


---

|  |  |
| --- | --- |
| Reference Count | 6 |


---

|  |  |
| --- | --- |
| Mechanism | Unknown |


---

|  |  |
| --- | --- |
| Reference:0 || Sentence | "In contrast with the results for serum iron, the levels of ferritin, total iron binding capacity (transferrin), zinc, copper and coeruloplasmin were not altered in any subject during the insulin infusion or at 24 h following discontinuation of the infusion." |
| PMID | 3548194 |
| Year | 1987 |
| Species | Human |
| Journal | Acta Endocrinol (Copenh) |
| RefScore | 1 |
| Source | PArchNLP |
  |
|  |  |


---

|  |  |
| --- | --- |
 Reference:1 || Sentence | "The serum zinc, vitamin A, albumin, copper and retinoid-binding protein content was measured in 27 patients with benign prostatic hyperplasia and 19 patients with carcinoma of the prostate." |
| PMID | 6194843 |
| Year | 1983 |
| Species | Human |
| Journal | Br J Urol |
| RefScore | 0 |
| Source | PArchNLP |
  ||  |  |


---

|  |  |
| --- | --- |
 Reference:2 || Sentence | "Nickel, copper, and zinc complexes of isatin (H(2)L(1)) and N-methylisatin 3-picolinoyl hydrazone (HL(2)), were synthesized and characterized by means of spectroscopic techniques." |
| PMID | 14729311 |
| Year | 2004 |
| Species | Human |
| Journal | J Inorg Biochem |
| RefScore | 0 |
| Source | PArchNLP |
  ||  |  |


---

|  |  |
| --- | --- |
 Reference:3 || Sentence | "We have investigated the possible interactions between the dietary intake of copper, zinc, or aluminum and the activities of the enzymes dopamine-beta-hydroxylase (DBH, EC.1.14.2.1) and phenylethanolamine-N-methyltransferase (PNMT, EC.2.1.1.28) in the male rat brain." |
| PMID | 6890193 |
| Year | 1982 |
| Species | Rat |
| Journal | Neurotoxicology |
| RefScore | 0 |
| Source | PArchNLP |
  ||  |  |


---

|  |  |
| --- | --- |
 Reference:4 || Sentence | "The glial cells markers glial fibrillary acidic protein and vimentin, and serum albumin having a capacity for binding of zinc and copper were detected by immunofluorescence histochemistry." |
| PMID | 8377517 |
| Year | 1993 |
| Species | Rat |
| Journal | J Neurosci Methods |
| RefScore | 1 |
| Source | PArchNLP |
  ||  |  |


---

|  |  |
| --- | --- |
 Reference:5 || Sentence | "Maternal and fetal tissues were assessed for copper concentrations, the activities of the cuproenzymes copper, zinc superoxide dismutase and ceruloplasmin, and the copper binding protein metallothionein." |
| PMID | 8118109 |
| Year | 1993 |
| Species | Rat |
| Journal | Reprod Toxicol |
| RefScore | 1 |
| Source | PArchNLP |
  |


---

|  |  |
| --- | --- |
